# Recurrence of Mitotically Active Cellular Fibroma of the Ovary

**DOI:** 10.1155/2009/803062

**Published:** 2009-01-12

**Authors:** Dario Bucella, Jean-Frédéric Limbosch, Frédéric Buxant, Philippe Simon, Isabelle Fayt, Vincent Anaf, Jean-Christophe Noël

**Affiliations:** ^1^Département de Gynécologie, Hôpital Universitaire Erasme, Route de Lennik 808, 1070 Brussels, Belgium; ^2^Département de Pathologie, Hôpital Universitaire Erasme, Route de Lennik 808, 1070 Brussels, Belgium

## Abstract

*Background*. 10% of ovarian fibromatous tumours typically exhibit increased cellularity, mitotic activity, and less frequently nuclear atypia. Therefore, the classification within the group of fibromatous tumours may represent some difficulties, thus, one or several of these features should appear. *Case*. We introduce the clinical and pathologic features based on one case of recurrence of a mitotically active cellular ovarian fibroma (MACF) in the pararectal fossa. This recurrence took place six years after primary surgery. Macroscopically, the tumour was firm, fibrous, well delimited, yellow-white without gross necrosis. On microscopic examination, it was composed of a densely cellular proliferation of fibrolastic-like cells with bland nuclear features and arranged in a fascicular pattern. There was no sign of significant atypia or necrosis. *Conclusion*. Recently, this case is the first report of a recurrence of MACF, following primary surgery with no tumoral rupture or surgical difficulty. The clinical outcome of ovarian cellular fibromas (CFs) and MACFs is typically uneventful. This case, however, strongly suggests maintaining a long-term clinical follow-up even though the principal tumour was surgically treated without tumour rupture or in the absence of adherence or any surgical difficulty.

## 1. Case Report

A 65-year-old woman looked for assistance to the gynecological consultation due to recent pelvic pain. Five years earlier, she went through a total hysterectomy with bilateral salpingoophorectomy for a benign ovarian mass described as an ovarian fibroma which eventually happened to be 10 cm long.

The gynecological exam did not show any mass, the vaginal cuff was soft and not tender. A vaginal ultrasound and a CT scan highlighted a pelvic mass of 10 cm in the pouch of Douglas and revealed no evidence of adenopathy. A laparoscopic exploration of abdominal cavity was performed. This pointed out a large mass in the Douglas while no other lesion inside the cavity was seen. Subsequently, the mass was resected and properly extracted in an endobag. Due to its very low position in the pelvis and to the hemorrhage, the resection could not be completed. Macroscopically, the tumour (12 × 10 × 9 cm) was firm, fibrous, well delimited, yellow-white without gross necrosis. On microscopic examination, it was made of a densely cellular proliferation of fibrolastic-like cells with bland nuclear features and arranged in a fascicular pattern. No significant atypia or necrosis could be observed. The tumoral cells were highly immunoreactive for vimentin and focally for alpha-inhibin, actin and CD99. Desmin, h-caldesmon, CD10, HMB-45, and c-kit were negative. The mitotic index was estimated at 4 mitoses/10 HPFs and the ki-67 index at 9%. After reviewing the slides concerning the primitive ovarian tumour, these presented similar morphological and immunohistochemical features.

**Figure 1:**
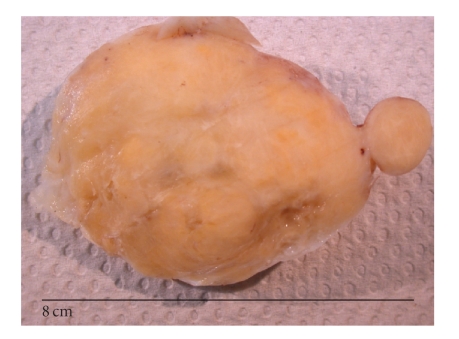
Recurring MECF macroscopic findings. Regular, lobulated, firm, and fibrous yellow-white tumours.

**Figure 2:**
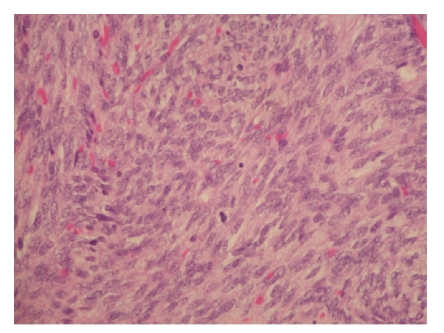
Microscopically, the tumour was composed of a dense proliferation of spindle cells, shaped cells with bland nuclei, and without no more mild cytological atypia. Note the presence of mitotic figures. Haematoxylin-eosin, X40.

Based on the above, it was hence decided to diagnose a recurrence of mitotically active cellular fibroma of the ovary (MACF). Six months later, the patient was diagnosed with a new recurrence which mass was 8 cm long and situated in the right pararectal region. The patient went though an MRI which later confirmed the recurrence of the mass. A laparotomy was then performed with a large resection of the pararectal recurrence (Figure 1). The pathological findings of this lesion were similar to the first recurrence description (Figure 2). It was decided to prescribe the patient with Tamoxifen (20 mg) on a daily basis and to schedule a long-term clinical follow-up. Six months after the surgery, no sign of new recurrence was noted.

## 2. Discussion

We present the clinical and pathologic features over one case of recurrence of a mitotically active cellular ovarian fibroma. This specific type of histological characteristic is rare. Ovarian stromal tumours composed of a pure proliferation of fibroblastic cells usually consist of fibromas, cellular fibromas (CFs), and hardly ever of fibrosarcomas. The majority of these neoplasms are benign fibromas with appreciable intercellular collagen, nonstriking cellularity, bland nuclei, and rare to absent mitotic figures (MFs).

About 10% of fibromatous tumours exhibit increased cellularity, mitotic activity, and less frequently nuclear atypia. It is then difficult to classify a case within the group of fibromatous tumours when one or several of these features exist.

In 1981, Prat and Scully suggested histological criteria for the distinction of CFs from fibrosarcomas based on a study including the analysis of 17 different cases [1]. This distinction was based on cellular proliferations of fibroblasts, nuclear atypia, mitotic count of 3 or fewer MFs per 10 high-power fields (MFs/10 HPFs) and malignant potential. For these authors, fibrosarcoma often exhibits moderate to severe nuclear atypia, mitotic counts of 4 or more MFs/10 HPFs, and a clinically malignant course. The opposite features were typical of CFs.

More recently, a study from Irving et al. reviewed 75 cases of ovarian CFs [2]. They all had poor features (cellular proliferations of fibroblasts, nuclear atypia, and a clinical malignant potential behaviour) except for the presence of significant mitotic rate. Thirty five cases presented 0 to 3 mitoses/10 HPFs and were classified as CFs. The mean age of patients was 51 years. The following 40 cases presented ≥4 mitoses/10 HPFs and were classified as MACFs. The mean age was 41 years.

No data about follow-up were highlighted for patients presenting CFs though these happened to be mentioned with regard to patients presenting MACFs. Finally, this study did not refer to any recurrence for patients with MACFs.

Since the first study published by Prat and Scully in 1981, a number of ovarian fibrosarcomas with a benign clinical course have been reported in the literature in which the diagnosis was based primarily or exclusively on a mitotic rate of 4 or more MFs/10 HPFs [3–11]. In those papers, only the 4 MFs/10 HPFs were taken into account to make the difference between CFs and fibrosarcomas. The later classification introduced in 2006 by Irving et al. of CFs was based on mitotic rate but also insisted on the fact that other histological and clinical features might explain the multitude of reports of ovarian fibrosarcomas with no recurrence after surgery. Irving et al. reviewed and reclassified those reports into MACFs. Recently, a new report of an MACF showed no evidence of recurrence after 1-year follow-up after surgery [12].

According to this classification, review of literature shows that only 2 cases of recurrence of MACFs have been reported [1]. Both patients with recurrences died in the following years. The first patient presented a primary ovarian tumour adherent to the pelvic wall and omentum, and the patient died of recurrent disease 2.75 years later. During primary surgery for the second patient, the ovarian tumour was ruptured. The patient died of unrelated causes and autopsy later confirmed the existence of a recurrent tumour.

Our case shows a recurrence 5 years following primary surgery. The first surgical intervention was performed without any problem hence no adhesiolysis was necessary and the ovarian tumour was extracted without rupture. Our report is the first to present a late recurrence of MACF after primary surgery which was previously performed with no tumoral rupture or surgical difficulty.

The clinical outcome of ovarian CFs and MACFs is typically uneventful, but this report strongly suggests long-term clinical follow-up even if primary tumour was surgically treated without rupture or in absence of adherence or surgical difficulty.
